# Comparison of GATK and DeepVariant by trio sequencing

**DOI:** 10.1038/s41598-022-05833-4

**Published:** 2022-02-02

**Authors:** Yi-Lin Lin, Pi-Chuan Chang, Ching Hsu, Miao-Zi Hung, Yin-Hsiu Chien, Wuh-Liang Hwu, FeiPei Lai, Ni-Chung Lee

**Affiliations:** 1 Department of Medical Genetics, National Taiwan University Hospital, 8 Chung-Shan South Road, Taipei, 10041 Taiwan; 2 Google Inc., 1600 Amphitheatre Pkwy, Mountain View, CA 94043 USA; 3 Graduate Institute of Biomedical Electronics and Bioinformatics, National Taiwan University, No. 1, Sec. 4, Roosevelt Rd, Taipei, 10617 Taiwan; 4 Department of Pediatrics, National Taiwan University Hospital, 8 Chung-Shan South Road, Taipei, 10041 Taiwan

**Keywords:** Computational biology and bioinformatics, Genetics, Molecular medicine

## Abstract

While next-generation sequencing (NGS) has transformed genetic testing, it generates large quantities of noisy data that require a significant amount of bioinformatics to generate useful interpretation. The accuracy of variant calling is therefore critical. Although GATK HaplotypeCaller is a widely used tool for this purpose, newer methods such as DeepVariant have shown higher accuracy in assessments of gold-standard samples for whole-genome sequencing (WGS) and whole-exome sequencing (WES), but a side-by-side comparison on clinical samples has not been performed. Trio WES was used to compare GATK (4.1.2.0) HaplotypeCaller and DeepVariant (v0.8.0). The performance of the two pipelines was evaluated according to the Mendelian error rate, transition-to-transversion (Ti/Tv) ratio, concordance rate, and pathological variant detection rate. Data from 80 trios were analyzed. The Mendelian error rate of the 77 biological trios calculated from the data by DeepVariant (3.09 ± 0.83%) was lower than that calculated from the data by GATK (5.25 ± 0.91%) (p < 0.001). DeepVariant also yielded a higher Ti/Tv ratio (2.38 ± 0.02) than GATK (2.04 ± 0.07) (p < 0.001), suggesting that DeepVariant proportionally called more true positives. The concordance rate between the 2 pipelines was 88.73%. Sixty-three disease-causing variants were detected in the 80 trios. Among them, DeepVariant detected 62 variants, and GATK detected 61 variants. The one variant called by DeepVariant but not GATK HaplotypeCaller might have been missed by GATK HaplotypeCaller due to low coverage. OTC exon 2 (139 bp) deletion was not detected by either method. Mendelian error rate calculation is an effective way to evaluate variant callers. By this method, DeepVariant outperformed GATK, while the two pipelines performed equally in other parameters.

## Introduction

Whole-exome sequencing (WES) by next-generation sequencing (NGS) has become an important tool in the diagnosis of inherited diseases^1^. An increasing number of medical centers consider WES first-line genetic testing. As NGS yields tens of thousands of short-read sequences, accurate variant calling is thus crucial for subsequent variant prioritization.

Several variant callers have been published^2–6^. Among them, GATK HaplotypeCaller is the most widely used. However, previous studies have also highlighted that different variant callers generated substantial disagreement^7–9^. DeepVariant won the PrecisionFDA Truth Challenge in 2016, demonstrating a higher accuracy in single-nucleotide polymorphism (SNP) detection than GATK^6,10^.

There are different ways to evaluate the performance of variant callers. Usually, reference DNA samples or FASTQ files are analyzed for this purpose. To evaluate variant callers on real-world data, in this study, we employed the Mendelian error rate and several other metrics to compare GATK and DeepVariant.

## Results

### General performance of the two pipelines

**Figure 1:**
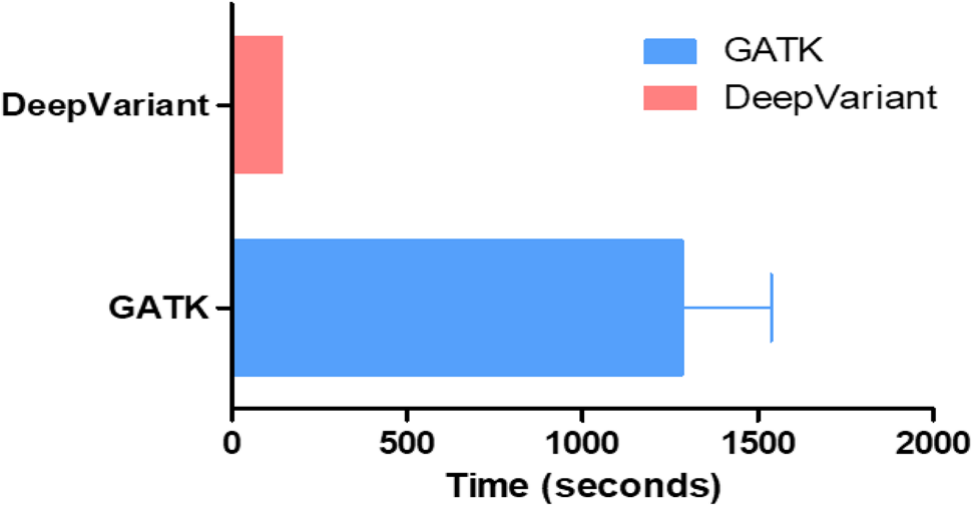
Variant calling execution time of GATK and DeepVariant for one typical trio exome analysis.

**Figure 2:**
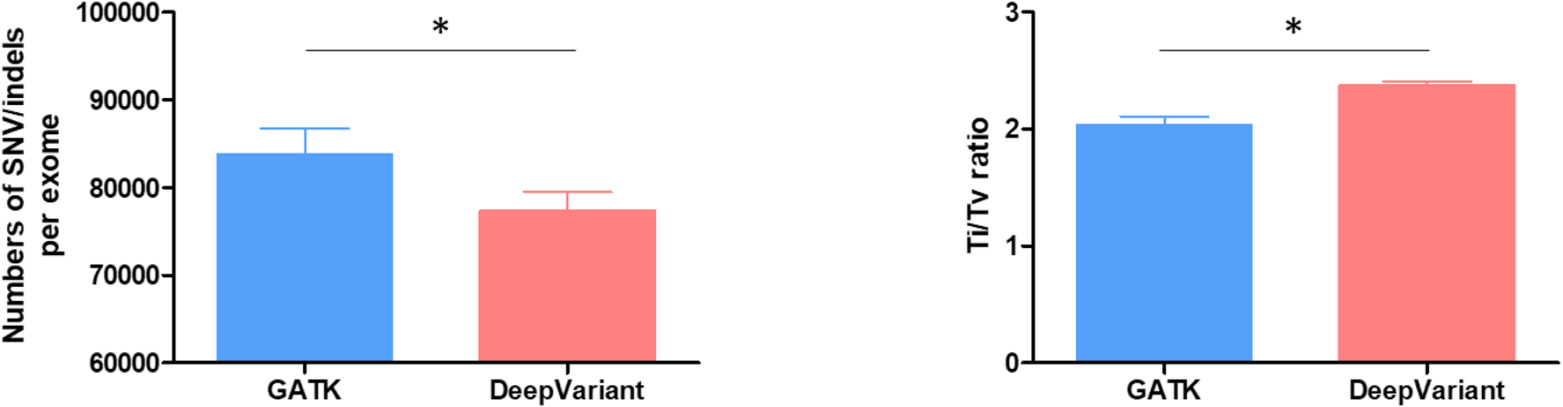
Numbers of SNVs/indels and Ti/Tv ratios from the 2 different pipelines (n = 77 trios).

**Figure 3:**
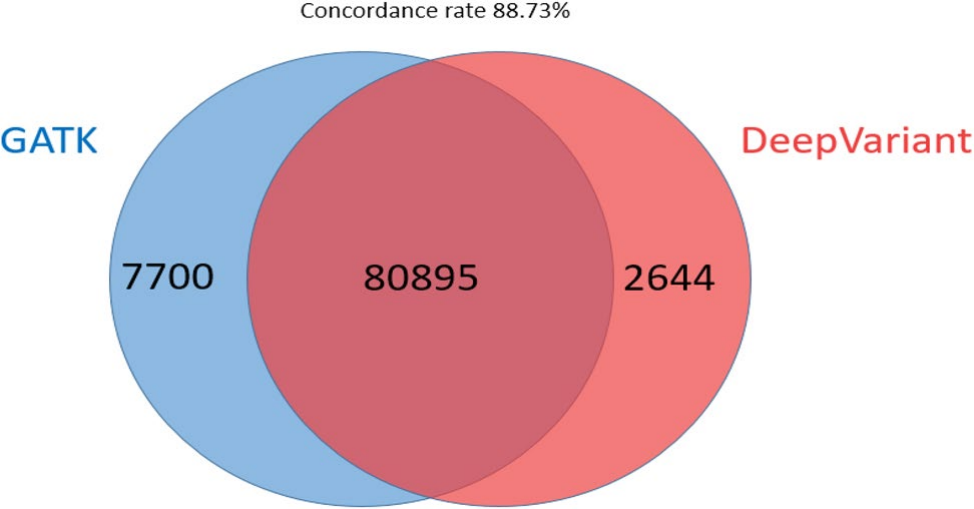
Venn diagram of variants identified by the two different pipelines (the number indicates the mean from 231 exomes; n = 77 trios).

The pipelines were executed on a server equipped with a 40 core CPU, 384 GB of RAM, and one V100 GPU. The execution time for one trio exome sequencing (patient, father, and mother) was 2 h 30 m for GATK and 1 h 30 m for DeepVariant (Fig. 1). The time required for variant calling was 3851 ± 253 s for GATK and 425 ± 0.6 s for DeepVariant (*p* = 0.046). Approximately 92,000–120,000 changes were identified for each patient. GATK called more single-nucleotide variants (SNVs), insertions, deletions, and indels, but DeepVariant gave a higher transition-to-transversion (Ti/Tv) ratio (2.04 for GATK and 2.38 for DeepVariant) (Table 1; Fig. 2). The Ti/Tv ratio is an important criterion for assessing the quality of SNV calling. For exome sequencing, the expected Ti/Tv ratio is approximately 3.0^11^. A higher Ti/Tv ratio usually indicates a higher accuracy of SNV calling. The concordance rate between GATK and DeepVariant was 88.73% (Fig. 3).

**Table 1:** Comparison of variant counts from the two pipelines (n = 77 trios).

	DeepVariant	GATK	P value
**Variant count**			
Total (SNVs + Ins + Del + Indels)	85,110 ± 2713	97,003 ± 4217	< 0.001
SNVs	77,338 ± 2103	83,723 ± 2973	< 0.001
Insertions	3786 ± 305	5641 ± 628	< 0.001
Deletions	3971 ± 339	7551 ± 666	< 0.001
Indels	14 ± 5	86 ± 26	< 0.001
**Ti/Tv ratio**			
SNVs	2.38 ± 0.025	2.04 ± 0.07	< 0.001

### Mendelian error rate

The Mendelian error rate of 80 trios was calculated from the data generated by the GATK and DeepVariant pipelines. Excluding the 3 families without 2 biological parents, the Mendelian error rate for the 77 biological trios was lower in DeepVariant (GATK vs. DeepVariant, 5.25 ± 0.91% vs. 3.09 ± 0.83%, p < 0.001; Table 2). In conjunction with the Ti/TV ratio results, DeepVariant yielded a higher Ti/Tv ratio, which indicated that DeepVariant has a higher accuracy. For the excluded three trio samples, each trio had only one biological parent, and the other was an unrelated person. The Mendelian error rate for these 3 families was high for both pipelines. From this result, we know that the Mendelian error rate could be used to identify true biological parents.

**Table 2:** Comparison of Mendelian error rates of variants from the two different pipelines.

	Mendelian error rate %		
	GATK	DeepVariant	P value
**Both biological parents (n = 77)**			
Mean ± SD (%)	5.25 ± 0.91	3.09 ± 0.83	< 0.001
Range (%)	3.84–8.72%	1.93–5.59%	
**One nonbiological parent (n = 3)**			
WES-A	19.84	18.94	
WES-B	19.05	18.09	
WES-C	19.71	18.84	

### Diagnostic yield of trio exome sequencing analysis for pediatric patients

We previously published the results for family 1–40 analyzed with GATK version 3.5 (Wu et al. 2019). In this study, we analyzed the same 40 families plus an additional 40 families using GATK version 4.1.2.0 and DeepVariant version 0.8.0. Following the same analysis procedures previously established for variant annotation and filtering (Wu et al. 2019), the same 21 variants were found in the first 40 families; 42 more variants related to the phenotypes of the patients were found in family 41–80. Overall, 40 out of 80 families received a diagnosis, achieving a diagnostic rate of 50% (Table S1). Among the 40 families with a diagnosis, 11 (27.5%) carried autosomal dominant mutations, 24 (60.0%) carried autosomal recessive mutations, and 5 (12.5%) carried X-linked mutations. For variant calling comparison, GATK identified 61 of the 63 variants (96.82%), while DeepVariant identified 62 variants (98.41%). The one variant that was differentially identified, NM_001101426.3: c.164G>A (p.Gly55Glu), which was called by DeepVariant, showed a quality score of 37.5. The reason why the GATK pipeline failed to call this variant might be due to its low coverage, with a read depth of 4. The other variant that was picked up by neither GATK nor DeepVariant was an *OTC* exon 2 deletion (139 bp). This miss was expected, as both tools are designed to call small variants (< 50 bp) but not large deletions.

## Discussion

In this study, we performed a real-world comparison of GATK and DeepVariant in trio exome samples. Our goal was to understand the applicability and performance of DeepVariant in analyzing patient samples. Our approach included a comparison of the execution time, variant calling accuracy, Ti/Tv ratio, and Mendelian error rate. Our data showed that DeepVariant had a shorter execution time and a smaller Mendelian error rate. DeepVariant made fewer calls, but with a lower false positive rate in variants failing Sanger sequencing and a lower false negative rate in identifying variants of interest. This finding in clinical samples concurred with recent applications in research cohorts^12^ and evaluations using simulated and gold-standard personal exome data^8,10^. In addition, we observed that the Ti/Tv ratio was higher in DeepVariant than GATK in the joint genotyping algorithm, which is in concordance with a recent report^12^.

It has been demonstrated that when used in joint genotyping, DeepVariant had better genotype quality (GQ) score calibration than GATK both in sequence-covered regions and by variant type^12^. Although variant quality normalization by read depth (quality of depth; QD) has been noted as the most important feature for discriminating true variants from artifacts in GATK calls for large-scale analysis, GQ is more informative for single-sample evaluation^12,13^.

There are several ways to perform variant caller comparisons. One method is to use gold-standard reference samples, such as HG001 or HG002^2,7,9^, for comparison because the true variants are known and verified. Testing trio cases by calculating the Mendelian error rate is another method that can demonstrate accuracy by comparing variants from parents. Compared to singleton analysis, checking the Mendelian error rate may be a more realistic way to understand the performance of variant calling. Liang et al. performed an analysis on the NA12878 trio (HG002, HG003, HG004) for the detection of de novo SNVs using GATK, RTC, and VarScan^14^. They found that GATK had better performance in identifying de novo SNVs in low GC-content regions, while RTG had a higher error rate in high GC-content regions^14^. In our observation, DeepVariant had better calling performance in the low coverage region than GATK. Since there was only one disease-causing variant with low coverage, further data comparison in low coverage regions will elucidate the differences between these two callers.

During comparison, we noticed that the quality scores of DeepVariant could not be linearly correlated with quality scores from GATK (Fig. S1). Even though DeepVariant has better calibrated quality scores, this discrepancy still presents a challenge for integration because some of our downstream applications were trained using GATK quality scores. To overcome this issue, establishment of DeepVariant-specific criteria for downstream application will be needed.

One limitation of this study is the relatively small sample size. However, our data are consistent, and the standard deviation is small, indicating that our findings are correct. Another limitation of this study is that we did not use gold-standard reference samples for comparison. This is because the majority of studies have already used reference samples; therefore, here, we reported our real-world experience to share first-hand information.

## Conclusions

Compared to GATK, DeepVariant had a shorter execution time and higher accuracy for clinical samples.

## Methods

### Subjects

Eighty patients suspected of having Mendelian disorders were referred for WES testing from June 2017 to May 2019. A total of 240 peripheral blood samples were collected. Two hundred thirty-seven samples were from the patients plus their biological parents (trio). As blood was unavailable for one of the parents in family 16, 74, and 76, unrelated healthy individuals whose sex matched the missing parent were used. Clinical data were provided by the referring physician. Genomic DNA was extracted with a QIAamp DNA Mini Kit (QIAGEN). In short, two hundred microliters of blood samples were used, and DNA was eluted in a volume of 100 µl. The quality and concentration of the extracted DNA samples were determined with a NanoDrop, and the samples were stored at 4 °C. This study was approved by the Institutional Review Board of National Taiwan University Hospital (IRB Nos. 201703073RINB and 20190057RIND). All methods were performed in accordance with relevant guidelines and regulations. Written informed consent was obtained from each family who participated in the study.

### Exome sequencing and variant calling

**Figure 4:**
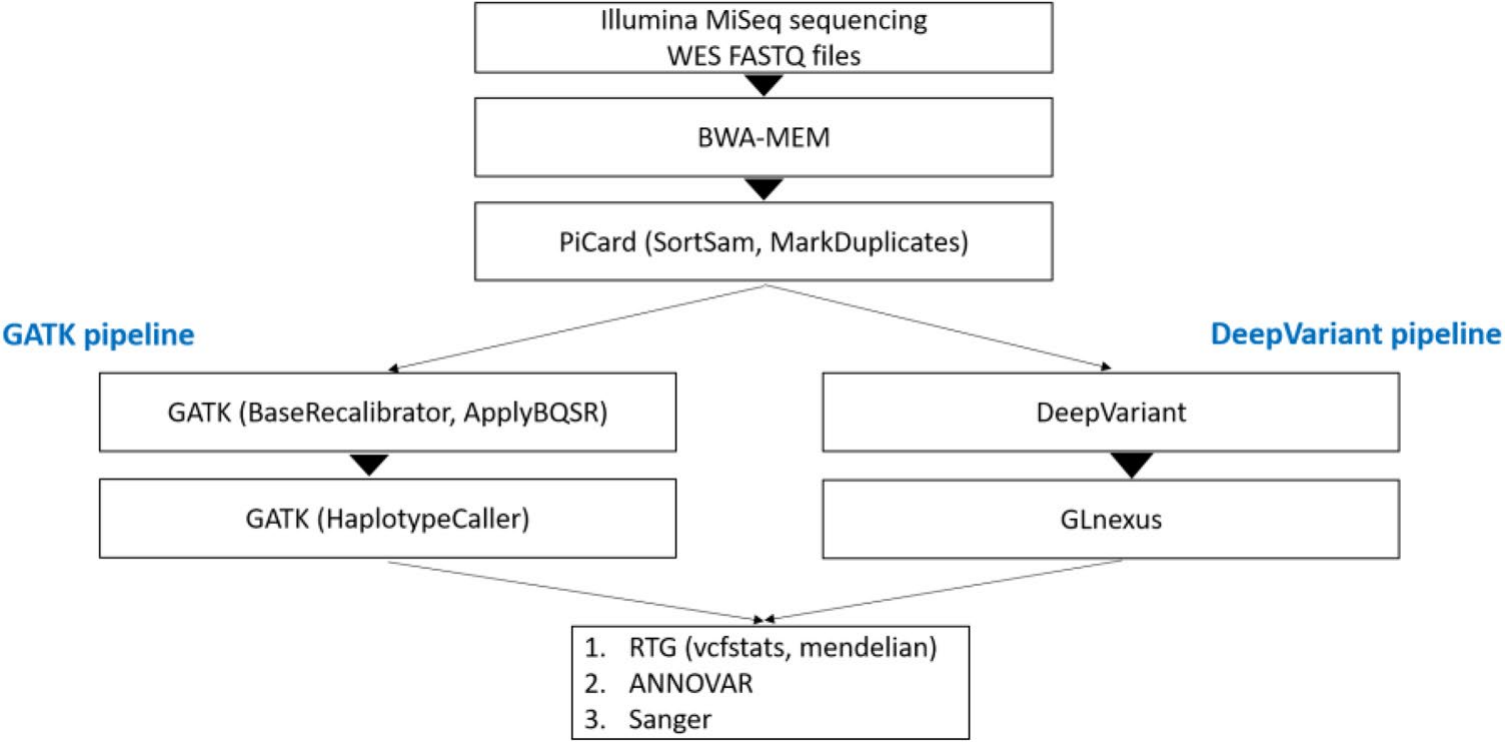
Algorithm of the bioinformatics pipelines.

Library preparations and sequencing were performed one trio at a time. Exome capture was carried out with a TruSeq DNA Exome Kit (Illumina), covering 214,405 exons with a total size of approximately 45 Mb. The captured libraries were then sequenced with a NextSeq 500 sequencer (Illumina) to produce 2 × 75 bp reads. Raw sequencing reads were converted to standard fastq format using bcl2fastq2 conversion software v2.20 (Illumina). Quality control of the raw reads was performed with FastQC. For variant calling, two different tools were used (Fig. 4), GATK and DeepVariant. The GATK pipeline was based on the best practices workflow for germlines established by the Broad Institute (https://gatk.broadinstitute.org/hc/en-us/articles/360035535932-Germline-short-variant-discovery-SNPs-Indels). Briefly, reads were aligned to the reference genome using BWA-MEM. The human reference genome used here was GRCh37. Picard tools were then used to sort and mark PCR duplicate reads, generating BAM files. GATK version 4.1.2.0 was used to recalibrate BAM files with BaseRecalibrator and ApplyBQSR and to generate VCF and GVCF files with HaplotypeCaller. DeepVariant analysis was performed according to the online instruction for germline variant calling with Illumina whole-exome data (https://github.com/google/deepvariant). In summary, after installing DeepVariant version 0.8.0 Docker, VCF and GVCF files were created with one single command using the BAM files processed by Picard. The GVCF files created from each pipeline were combined separately for each trio to calculate the Mendelian error rate. The VCF files were used for annotation and analysis.

### Variant annotation and filtering

Variant annotation was performed by wANNOVAR (http://wannovar.wglab.org). Variants were then filtered with various criteria: (1) quality score for GATK > 200 and DeepVariant > 25, (2) pass filter, and (3) allele frequency < 0.05 in the population database, such as Exome Aggregation Consortium (ExAC), 1000 Genomes Project, ESP6500si, and dbSNP. Variants located in introns, 3' or 5' untranslated regions (UTRs), upstream, downstream, or noncoding regions, or those that would cause synonymous mutations and in-frame indels were also not included, as the majority of variations occurring in these regions have insufficient evidence. Filtered variants were subsequently prioritized according to the patient’s clinical diagnosis and phenotypes. The probable mutations were evaluated in the order of the following consequences: initiation codon, stop loss/gain, splice site, frameshift, and missense. Candidate variants were classified into likely pathogenic (LP)/pathogenic (P), likely benign (LB)/benign (B), or variants of unknown significance (VUSs) according to American College of Medical Genetics and Genomics (ACMG) guidelines^15^. We looked at databases such as the Human Gene Mutation Database (HGMD) and Online Mendelian Inheritance in Man (OMIM), the effect of each change on the protein and splice site, and different scores such as nucleotide and amino acid conservation scores, SIFT, and PolyPhen-2^16,17^. After variant prioritization, genotype–phenotype correlation was reviewed by physicians. Selected disease-causing variants were selected for Sanger sequencing. Assessment of the quality of sequencing and the variants was performed with Integrative Genomics Viewer (IGV). The coverage of exons in each gene was calculated. Those that did not have adequate coverage were highlighted.

### Verification by Sanger sequencing

Sanger sequencing was carried out to verify the variants detected by NGS. Sixty-three disease-causing variants from each family were chosen based on genotype–phenotype correlations after variant filtering (Wu et al. 2019) and ranking by our in-house AI_driven module (manuscript in submission). Sanger sequencing was performed on variants found in family 1 to family 32. After that, our laboratory established internal criteria for Sanger sequencing of candidate variants as (1) SNVs with GATK quality scores less than 500 and (2) small indel variants. Specific primers were designed using Primer3 v.0.4.0 (http://bioinfo.ut.ee/primer3-0.4.0/) and purchased from Bio-Protech. Genomic DNA was amplified by touch-down PCR using a GeneAmp PCR System (Applied Biosystems). For PCR products with GC contents exceeding 59%, 5% DMSO was added to the mixture. Thermal cycles were performed with 1 cycle of 94 °C for 5 min; 14 cycles of touch-down PCR with 94 °C for 30 s, touch-down annealing temperature for 30 s, and 72 °C for 30 s; 25 cycles of 94 °C for 30 s, 53 °C for 30 s, and 72 °C for 30 s; and finally, 1 cycle at 72 °C for 5 min. The annealing temperature of the touch-down cycles started at 60 °C initially and was decreased by 0.5 °C each cycle, ending at 53 °C. Agarose gel electrophoresis was used to confirm PCR products, and all primer sets produced a single band. The PCR products were purified with a Gel/PCR DNA Fragments Extraction Kit (Geneaid), followed by direct sequencing using an ABI Prism Big Dye Dideoxy Chain Terminator Cycle Sequencing Kit and an ABI Prism 3100 genetic analyzer (Applied Biosystems).

### Statistical analysis

The results from the GATK and DeepVariant pipelines were evaluated with RTG tools (https://github.com/RealTimeGenomics/rtg-tools). In addition to the number of variants called, the Ti/Tv ratio was also calculated by RTG-vcfstats and used as an indicator of potential sequencing error. Mendelian violation of the trio data was detected with RTG-mendelian. Additionally, concordance analysis between the pipelines was performed with SPSS v17. The significance was determined by Student’s t test.

### Ethics approval and consent to participate

Written informed consent was obtained from all participants.

### Consent for publication

Written informed consent was obtained from all participants.

## Supplementary Information

Supplementary Information.

## Data Availability

The datasets generated and/or analyzed during the current study are not publicly available due to the anonymity of the participants but are available from the corresponding author on reasonable request.
