# Supplementary material for: Comparison of GATK and DeepVariant by trio sequencing
Source: Sci Rep. 2022 Feb 2;12:1809. doi: 10.1038/s41598-022-05833-4 (PMC8810758; doi:10.1038/s41598-022-05833-4)
Supplement: Supplementary file 1 — Supplementary Information. [file 41598_2022_5833_MOESM1_ESM.pdf]

Figure S1 Correlation of quality scores between GATK and DeepVariant. In one typical whole exome sequencing.

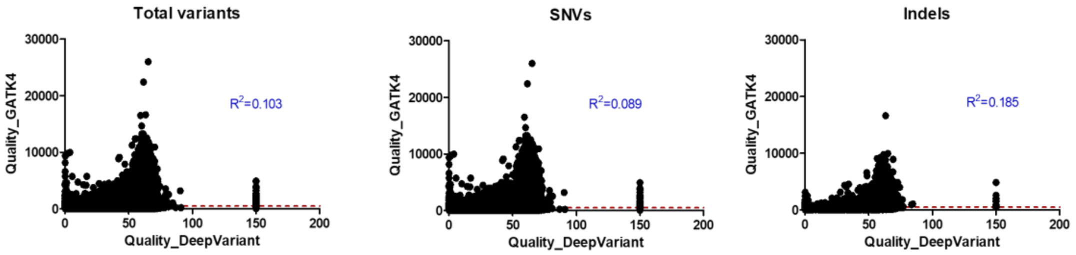

Table S1 List of all 40 positive patients enrolled in this study

Family	Gene	Variant 1	Variant 2	Molecular diagnosis	Inheritance
1	CD3E	11:118183354_ 118183359del	11:118184505_ 118184506insTGCTGGT	Immunodeficiency 18	AR
2	EIF2B5	3:183855493C>T	3:183858252G>T	Vanishing white matter syndrome	AR
3	COQ4	9:131088128G>A	9:131088128G>A	Primary coenzyme Q10 deficiency-7 (COQ10D7)	AR
7	MOCS2	5:52405544G>A	5:52405544G>A	Molybdenum cofactor deficiency of complementation group B (MOCODB)	AR
8	ISPD	7:16317782A>G	7:16460784C>T	Congenital muscular dystrophy-dystroglycanopathy with brain and eye anomalies (type A7; MDDGA7)	AR
9	NGLY1	3:25775392G>A	3:25778867G>A	Congenital disorder of deglycosylation (CDDG)	AR
10	COQ4	9:131088128G>A	9:131088128G>A	Primary coenzyme Q10 deficiency-7 (COQ10D7)	AR
11	PEX1	7:92122349A>G	7:92123671A>G	Peroxisome biogenesis disorder 1C (PBD1C)	AR
14	PITX2	4:111542336dupT	.	Axenfeld-Rieger syndrome type 1	De Novo
15	KCNQ5	6:73821106C>T	.	Mental retardation, autosomal dominant 46	De Novo
16	RYR2	1:237798237C>T	.	Ventricular tachycardia, catecholaminergic polymorphic, 1	NA
18	SCN8A	12:52180362A>G	.	Epileptic encephalopathy, early infantile, 13	De Novo
20	PEX7	6:137146400delT	6:137191088C>T	Rhizomelic chondrodysplasia punctate type 1	AR
22	C3	19:6677998C>G	19:6692982C>T	atypical Hemolytic uremic syndrome plus autoimmune disease	AR

25	TRPC6	11:101323793C>T	.	Glomerulosclerosis, focal segmental, 2	AD father
27	TNNI3	19:55665415T>C	.	Cardiomyopathy, familial restrictive, 1	De Novo
28	CPS1	2:211469884T>G	2:211525245C>T	Carbamoylphosphate synthetase I deficiency	AR
32	SLC22A5	5:131721127C>T	5:131721127C>T	Primary carnitine deficiency	AR
34	PAH	12:103237461C>T	12:103246707C>T	Phenylketonuria	AR
36	RAG1	11:36597711G>T	.	Omenn syndrome	AR
38	EYA1	8:72182009_ 72182012del	.	Branchiootorenal syndrome 1, with or without cataracts	AD father
41	IFT122	3:129180097C>T	3:129183597T>G	Cranioectodermal Dysplasia	AR
46	OTC	X:38240594G>A	.	Ornithine transcarbamylase deficiency	XL
48	OTC	Exon2 del	.	Ornithine transcarbamylase deficiency	XL
49	TBX1	22:19753938C>T	.	DiGeorge syndrome	De Novo
50	TARS2	1:150463159C>G	1:150471505G>A	Combined oxidative phosphorylation deficiency 21	AR
51	SCN2A	2:166237211T>C	.	Epileptic encephalopathy, early infantile, 11	De Novo
54	FGFR3	4:1806153C>A	.	Crouzon syndrome with acanthosis nigricans	De Novo
55	EARS2	16:23540932C>T	16:23540932C>T	Combined oxidative phosphorylation deficiency 12	AR
56	ABCD1	X:152991286C>T	.	Adrenoleukodystrophy	XL
57	AMT	3:49457783del CAAAGinsAC	3:49457787G>C	Glycine encephalopathy	AR
59	TALDO1	11:763382C>T	11:763470G>T	Transaldolase deficiency	AR
60	SMPD1	11:6413290C>G	11:6413290C>G	Niemann-Pick disease, type B	AR

64	KCNQ2	20:62076066G>C	.	Epileptic encephalopathy, early infantile, 7	De Novo
66	APOA1BP	1:156563742A>C	1:156563742A>C	Encephalopathy, progressive, early-onset, with brain edema and/or leukoencephalopathy	AR
67	SLC25A13	7:95818684_ 95818687del	7:95822344C>T	Citrullinemia, type II, neonatal-onset	AR
71	KLHL40	3:42730455A>C	3:42730521G>A	Nemaline myopathy 8, autosomal recessive	AR
73	DKC1	X:154004468C>G	.	Dyskeratosis congenita, X-linked	XL
74	MTM1	X:149787510G>A	.	Myotubular myopathy, X-linked	NA
77	PEX1	7:92129026_ 92129027del	7:92131228_ 92131229del	Peroxisome biogenesis disorder 1A (Zellweger)	AR

AD, autosomal dominant; AR, autosomal recessive; NA, not available because only one biological parent received the analysis; XL, X-linked
